# 

**Published:** 1869-05-01

**Authors:** 


### CHICAGO MEDICAL JOURNAL.

J. ADAMS ALLEN, M.D., LL.D., Editor. WALTER HAY, A.M., M.D., Associate Editor.

All letters for THE JOURNAL should be addressed to DR. J. ADAMS ALLEN, No. 71 Dearborn Street, Chicago, Ill.

TERMS—$3 PER ANNUM, IN ADVANCE.

$4 if not paid before 1st of July, in each year. Single Copies, 25 Cents.

### SPECIAL NOTICE.

*The publication of the* Chicago Medical Journal *having passed into the hands of the subscriber, all payments for subscription and accounts, either past or future, must be made to me, unless otherwise directed.*

*The* Editorial Management *will remain as heretofore, in the hands of* Dr. J. Adams Allen, *assisted by* Dr. W. Hay, *and to whom communications in his department should be addressed.*

J. WOLCOTT HOOKER.

*Chicago, May 1st.*

### BOOKS RECEIVED.

THE STRUCTURAL LESIONS OF THE SKIN: Their Pathology and Treatment. Illustrated. By HOWARD F. DAMON, A.M., M.D., Fellow of the Massachusetts Medical Society, etc., etc. Philadelphia: J. B. Lippincott & Co., 1869. Pp. 255.

A very acceptable and interesting contribution to the scientific history of this important class of diseases. We cordially commend it to our readers.

DESCRIPTIVE CATALOGUE OF THE PATHOLOGICAL MUSEUM OF THE PENNSYLVANIA HOSPITAL. By WILLIAM PEPPER, M.D., Physician to the Philadelphia Hospital, Curator and Pathologist to the Pennsylvania Hospital, etc., etc. Philadelphia: Published by the Board of Managers. For sale by Lindsay & Blakiston, 25 South Sixth Street, Philadelphia. Pp. 138.

A PRACTICAL TREATISE ON THE DISEASES OF WOMEN. By T. GAILLARD THOMAS, M.D., Professor of Obstetrics and the Diseases of Women and Children in the College of Physicians and Surgeons, New York, etc., etc., with two hundred and twenty-five Illustrations. SECOND EDITION, Revised and Improved. Philadelphia: Henry C. Lea, 1869. Pp. 647.

The favor in which this book is held by the Profession is evinced by the exhaustion of the first edition (a very large one), within six months from its publication; a well-deserved success.

### PAMPHL[illegible]TS RECEIVED.

SUPPLEMENT TO THE REPORT OF THE BOARD OF TRUSTEES OF THE MICHIGAN ASYLUM FOR THE INSANE, for the years 1867-8.

THE PART TAKEN BY NATURE AND TIME IN THE CURE OF DISEASES.

REPORT ON OBSTETRICS, read before the State Medical Society, session of 1868, by ABRAM SAGER, M. D.

THE INTERMARRIAGE OF RELATIONS, by NATHAN ALLEN, M.D.

ON THE TREATMENT OF PARALYSIS BY ELECTRIZATION, with an Explantion of a new galvainc apparatus, by A. D. ROCKWELL, M. D.

HALF YEARLY COMPENDIUM OF MEDICAL SCIENCE. Part 3d. January, 1869.

